# Effects of slaughter age and muscle type on meat quality characteristics of Eastern Anatolian Red bulls

**DOI:** 10.5194/aab-61-497-2018

**Published:** 2018-12-20

**Authors:** Sinan Kopuzlu, Nurinisa Esenbuga, Alper Onenc, Muhlis Macit, Mete Yanar, Sadrettin Yuksel, Abdulkadir Ozluturk, Necdet Unlu

**Affiliations:** 1 Department of Animal Science, College of Agriculture, Atatürk University, 25240, Erzurum, Turkey; 2 Department of Animal Science, College of Agriculture, Namık Kemal University, Tekirdag, Turkey; 3 Eastern Anatolian Agricultural Research Institute, Erzurum, Turkey

## Abstract

The effects of slaughter age and muscle type on meat quality properties of Eastern Anatolian Red (EAR) bulls (n=46) were investigated in the present study. Forty-six EAR bulls were slaughtered at 15, 17, 19, 25 and 27 months. Meat samples were taken from longissimus dorsi (LD) and gluteus medius (GM) muscles obtained from the carcasses at 24 h post-mortem. Meat color parameters were significantly affected by slaughter age. Older animals (19, 25 and 27 months of age) possessed higher $L^{*}$, $a^{*}$ and H values than those of younger animals (15 and 17 months of age). LD muscle had higher $L^{*}$ and $a^{*}$ values than those of GM muscles. The influences of slaughter age and muscle type on the proportions of dry matter, ether extract, crude ash and crude protein were not significant in the present study. The influence of slaughter age on the tenderness, acceptability, juiciness, number of chews and Warner–Bratzler shear (WBS) values were found to be significant. Tenderness, juiciness, flavor intensity and acceptability increased until 19 months of age, and then increase in age resulted in lower tenderness, flavor intensity and acceptability scores. Cooking yield significantly increased depending on the increase of age. WBS and cooking yield values from the LD were higher than that of the GM muscle. Textural characteristics such as hardness, springiness, cohesiveness, gumminess, chewiness and resilience were not affected by slaughter age, but muscles had a significant influence on hardness, gumminess, chewiness and resilience. In conclusion, most eating quality characteristics and color parameters were positively influenced by slaughter age.

## 1 Introduction

The changes in the world and Turkey meat markets over the past decade and the recruitment in the educational and economical conditions of most consumers have raised the demands related to the meat (Vieira et al., 2007). It is becoming increasingly obvious that consumers will continue to demand a leaner meat product in the market (Cross et al., 1984). Consumers all over the world are health conscious and demand high-quality meat products. Eventually, the success of any food product is determined by consumer acceptability, which is largely appointed by the perception of quality (Dransfield et al., 2003; Hoffman et al., 2003). Many factors influence meat quality, and all of them can be divided for simplicity into two categories: intrinsic factors such as breed, age, sex, slaughter weight, diet etc. and extrinsic factors such as management, slaughtering procedures, aging time etc. (Preziuso and Russo, 2004; Monsón et al., 2005).

Consumers prefer meat obtained from animals slaughtered at light weight or youthful beef because it is believed that the younger animals yield more tender meat than older, heavier animals (Vergara et al., 1999). Slaughter weight affects carcass and meat quality traits such as meat physiology, which concerns the properties and structure of muscle (Saňudo et al., 2004).

In eastern Turkey, Eastern Anatolian Red (EAR) is the most dominant native breed, comprising approximately 20.4 % of the indigenous breeds (Anonymous, 2015) and is predominantly used for meat production. EAR bulls are well adapted to the poor pasture, harsh climate and severe conditions. These are the characteristics of the hills and uplands of East Anatolia, where the sea level is 1300–2000 m, with an average winter temperature of -10 ${}^{\circ }$C and annual rainfall of 476 mm. There is no detailed investigation related to the influences of the slaughter age and weight on meat quality as well as texture, color, pH, chemical composition and sensory attributes of the beef from EAR cattle (Ozluturk et al., 2008; Unlu et al., 2008; Yuksel et al., 2012).

The aim of this study was to compare pH, color characteristics, Warner–Bratzler shear (WBS) force, sensory attributes, chemical compositions and texture characteristics of meat from longissimus dorsi (LD) and gluteus medius (GM) muscles of EAR bulls slaughtered at different ages or weights.

## 2 Materials and methods

### 2.1 Animals, diets and sampling

The study was carried out at the Eastern Anatolian Agricultural Research Institute, Erzurum, Turkey. Forty-six carcasses of male EAR cattle, reared on the same farm, fed on the same diet, and slaughtered at 15, 17, 19, 25 and 27 months of age were used as animal material. The animals used in this study were treated in accordance with the current regulations and standards issued by the Republic of Turkey Ministry of Agriculture and Forestry. The experimental procedures were approved by the Committee of Atatürk University Ethics and Animal Welfare Committee. The animals were individually fed and were fattened in a tethered barn. Cattle were fed on a ration consisting of concentrate and air-dry alfalfa. The average live weights of bull groups slaughtered at 15, 17, 19, 25 and 27 months of age were 270.0±16.36, 292.6±14.64, 338.5±13.36, 349.0±14.64 and 382.3±18.61 kg, respectively. Carcasses were chilled and stored 24 h post-mortem. Meat samples were taken from LD and GM muscles cut out from the carcasses 24 h post-mortem.

### 2.2 Meat physical and chemical parameters

pH value was evaluated on freshly cut surfaces of LD and GM muscles by direct probe using a SCHOTT, Lab Star pH meter. After slaughter, color properties were measured on LD and GM muscles 24 h after 30 min of exposure to the air. Minolta colorimeter device (CR-200, Minolta Co Osaka, Japan) was objectively used to measure CIELAB (Commission Internationale I'E Clairage) brightness ($L^{*}$), redness ($a^{*}$), yellowness ($b^{*}$), chroma ($C^{*}$) and hue (H) values on the LD and GM muscles (Aurand et al., 1987; Rödel, 1992). The muscle gobbets were cut perpendicular to the muscle fiber into two parts and utilized for chemical, sensory and textural analysis. AOAC (2006) was used to determine the dry matter, ether extract, crude protein and ash contents of meat samples from LD and GM muscles. The amount of crude protein was determined using Kjeldahl method as N×6.25.

### 2.3 Sensory analysis, texture profile and evaluation

To evaluate the sensory characteristics, meat samples were cooked in a plastic bag, in a water bath at 90 ${}^{\circ }$C until they reached an internal temperature of 70 ${}^{\circ }$C. Cooked samples were cut into two slices and subjected to sensory evaluation and WBS. Calculation of cooking yield was determined by dividing cooked weight by uncooked weight. To remove cooking drip, cooked samples were put on a paper towel for 5 min. The cooked LD and GM muscles were sliced into samples of approximately 10 g and then presented to a sensory panel consisting of nine experienced judges. The panel was formed to evaluate the cooked beef samples from LD and GM muscles in terms of tenderness, juiciness, flavor intensity and acceptability using nine-point hedonic scale (9 = extremely tender, 1 = extremely tough; 9 = extremely juicy, 1 = extremely dry; 9 = extremely strong beef flavor, 1 = extremely weak flavor; 9 = extremely high acceptability, 1 = extremely low acceptability). Number of chews before swallowing was also appointed by the panelists. Meat samples from different slaughter age groups were randomly presented to the panelist. Each sample was tested twice by each panelist. Mechanical assessment of meat samples cooled up to 20 ${}^{\circ }$C for tenderness was also determined using the WBS device (Esenbuga et al., 2009).

Following blast-freezing and storing (-18 ${}^{\circ }$C), slices were thawed (4 ${}^{\circ }$C) up to 2–5 ${}^{\circ }$C an internal temperature and cooked to 70 ${}^{\circ }$C – a final internal temperature in water bath (80 ${}^{\circ }$C). The analysis of texture profile (TPA) was performed on the meat samples (six cores 1.5 cm in diameter) using cross head speed of 300 mm min${}^{-1}$ and compression distance of 1 cm (Bourne, 1978). All of the measurements related to the samples were undertaken using a steel with 3.5 cm diameter sphere. All the measurements were performed using a Texture analyzer TA.TX2 (stable Micro Systems Ltd.) with a 50 kg load cell. Hardness, chewiness, cohesiveness and resilience parameters were obtained from the TPA curves.

**Table 1:** Effect of slaughter age and muscle type on pH and color parameters (mean ± SEM).

	Slaughter age (A)						Muscle (M)			Significance		
	15 months	17 months	19 months	25 months	27 months		GM	LD		A	M	A×M
pH	$5.44\pm 0.01^{b}$	$5.47\pm 0.01^{\mathrm{ab}}$	$5.48\pm 0.01^{a}$	$5.49\pm 0.01^{a}$	$5.49\pm 0.01^{a}$		5.44±0.01	5.48±0.01		${}^{*}$	${}^{**}$	${}^{*}$
Meat color												
$L^{*}$	$38.13\pm 0.39^{b}$	$38.47\pm 0.36^{b}$	$40.17\pm 0.36^{\mathrm{ab}}$	$41.42\pm 0.36^{a}$	$41.92\pm 0.41^{a}$		38.66±0.47	39.98±0.34		${}^{**}$	${}^{*}$	ns
$a^{*}$	$18.99\pm 0.49^{b}$	$19.46\pm 0.44^{b}$	$22.85\pm 0.44^{a}$	$22.88\pm 0.44^{a}$	$22.45\pm 0.52^{a}$		20.58±0.42	21.93±0.42		${}^{**}$	${}^{*}$	ns
$b^{*}$	$4.99\pm 0.29^{a}$	$5.02\pm 0.28^{a}$	$4.71\pm 0.28^{\mathrm{ab}}$	$4.82\pm 0.28^{\mathrm{ab}}$	$4.72\pm 0.31^{b}$		4.96±0.26	4.76±0.26		${}^{*}$	ns	ns
$C^{*}$	$14.75\pm 0.48^{a}$	$14.41\pm 0.45^{a}$	$11.68\pm 0.45^{b}$	$11.85\pm 0.45^{b}$	$11.79\pm 0.51^{b}$		13.51±0.45	12.19±0.45		${}^{*}$	ns	ns
$H^{*}$	$19.65\pm 0.64^{b}$	$20.05\pm 0.51^{b}$	$23.36\pm 0.51^{a}$	$23.32\pm 0.51^{a}$	$22.89\pm 0.91^{a}$		21.09±0.54	22.44±0.54		${}^{*}$	ns	ns

${}^{a,b}$ Means in rows with different superscripts are significantly different at p<0.05 or p<0.01; ns: non-significant (p>0.05); ${}^{**}$ p<0.01; ${}^{*}$ p>0.05; SEM: standard error of means.

## 3 Statistical analysis

Data related to pH, color characteristics, the sensory panel, chemical compositions and texture characteristics were analyzed using the GLM procedure of SPSS (2008) considering different slaughter age, muscle type and their interactions as main effects. A simple correlation among the sensory panel, chemical compositions, texture characteristics and shear force value were calculated. The Duncan test was used to compare mean values at a significance of p<0.05 (SPSS, 2008). The statistical model used for analysis of variance was as follows:

1 $$y_{ijk}=\mu +a_{i}+b_{j}+(ab{)}_{ij}+e_{ijk},$$

where $y_{ijk}$ is pH, color parameters, chemical compositions, sensory characteristics and texture parameters; μ is overall mean; $a_{i}$ is slaughter age; $b_{i}$ is muscle type; $(ab{)}_{ij}$ is the effect of interaction between slaughter age and muscle types; and $e_{ijk}$ is random error.

**Table 2:** Effect of slaughter age and muscle type on chemical composition, sensory characteristics and textural parameters of meat (mean ± SEM).

	Slaughter age (A)						Muscle (M)			Significance		
	15 months	17 months	19 months	25 months	27 months		GM	LD		A	M	A×M
Chemical composition												
Dry matter (%)	23.70±0.44	24.59±0.42	24.44±0.40	25.59±0.42	24.40±0.54		24.45±0.28	24.63±0.28		ns	ns	ns
Fat (%)	1.30±0.24	1.22±0.22	1.51±0.21	1.52±0.22	1.49±0.29		1.21±0.15	1.57±0.15		ns	ns	ns
Protein (%)	21.14±0.32	21.74±0.30	21.19±0.29	21.41±0.30	21.50±0.39		21.19±0.20	21.60±0.20		ns	ns	ns
Ash (%)	0.95±0.05	1.14±0.05	1.03±0.05	1.07±0.05	0.94±0.07		1.03±0.03	1.02±0.03		ns	ns	ns
Sensory characteristics												
Tenderness	$3.71\pm 0.25^{c}$	$5.22\pm 023^{\mathrm{ab}}$	$5.53\pm 0.23^{a}$	$5.19\pm 0.23^{\mathrm{ab}}$	$5.14\pm 0.32^{b}$		4.66±0.16	5.06±0.16		${}^{**}$	ns	${}^{*}$
Juiciness	$4.62\pm 0.21^{c}$	$4.84\pm 0.19^{\mathrm{bc}}$	$5.42\pm 0.19^{b}$	$5.11\pm 0.19^{\mathrm{bc}}$	$6.29\pm 0.27^{a}$		4.99±0.13	5.52±0.26		${}^{**}$	${}^{**}$	${}^{*}$
Flavor intensity	$5.39\pm 0.20^{b}$	$6.05\pm 0.18^{a}$	$6.57\pm 0.18^{a}$	$6.18\pm 0.18^{a}$	$6.20\pm 0.25^{a}$		5.80±0.13	6.19±0.13		${}^{**}$	${}^{*}$	${}^{*}$
Acceptability	$5.05\pm 0.22^{c}$	$5.99\pm 0.19^{\mathrm{ab}}$	$6.28\pm 0.19^{a}$	$5.98\pm 0.19^{\mathrm{ab}}$	$5.73\pm 0.27^{b}$		5.64±0.14	5.82±0.14		${}^{**}$	ns	ns
NCBS	$54.80\pm 2.50^{a}$	$37.49\pm 2.25^{b}$	$37.17\pm 2.45^{b}$	$41.36\pm 2.25^{b}$	$38.47\pm 3.16^{b}$		41.46±1.59	42.65±1.59		${}^{**}$	ns	ns
Textural parameters												
Cooking yield (%)	$62.82\pm 1.87^{b}$	$62.65\pm 1.77^{b}$	$64.54\pm 1.69^{\mathrm{ab}}$	$64.74\pm 1.77^{\mathrm{ab}}$	$66.55\pm 2.28^{a}$		61.54±1.19	67.78±1.19		${}^{*}$	${}^{**}$	ns
WBS	$10.32\pm 0.29^{a}$	$7.67\pm 0.27^{b}$	$6.71\pm 0.25^{c}$	$6.39\pm 0.28^{c}$	$5.39\pm 0.28^{d}$		7.41±0.18	7.99±0.18		${}^{**}$	${}^{*}$	${}^{**}$

${}^{a,b,c,d}$ Means in rows with different superscripts are significantly different at p<0.05 or p<0.01; ns: non-significant (p>0.05); ${}^{**}$ p<0.01; ${}^{*}$ p>0.05; SEM: standard error of means; NCBS: number of chews before swallowing; WBS: Warner–Bratzler shear value.

## 4 Results and discussion

Many consumers evaluate the meat quality and acceptability on account of pH and color from the meat quality criteria. Higher values with respect to pH were determined for meat samples obtained from the muscles of older animals (p<0.05); compared to LD muscle, pH values were higher than those of GM muscles (p<0.01). Significant interactions on account of these traits between muscle type and slaughter age were detected in the present study. Although it was significant, the differences in pH values measured 24 h after slaughter among the age groups were found to be low. Though the pH values changed between 5.35 and 5.60 values, this situation did not induce increased risk of a negative influence on meat quality. The important problems related to meat pH${}_{24}$ mentioned above are a dark red color, lower tenderness and increased water-holding capacity (Bures and Barton, 2012; Mach et al., 2008). Tenderness, being one of the most important factors affecting the taste of meat, is directly related to pH. The tenderness of the meat occurs at high and low pH. While high-pH meats are not desirable because they are elastic and darken in color, low-pH meat is desired by consumers as they are pale and juicy (Yakan, 2008).

Meat color parameters were affected by slaughter age (p<0.05; p<0.01) (Table 1). Older animals (19, 25 and 27 months of age) had higher $L^{*}$, $a^{*}$ and H values than those of younger animals (15 and 17 months). In terms of muscles, meat color parameters except for $L^{*}$ and $a^{*}$ were not significant. LD muscle had higher $L^{*}$ and $a^{*}$ values than GM muscles (p<0.05). Meat derived from LD muscle appears significantly lighter colored (higher $L^{*}$ value; p<0.05). Moreover, LD muscle was redder (higher $a^{*}$ value; p<0.05) than GM muscle. The influences of slaughter age on the meat color parameters were significant (p<0.05; p<0.01). Similar results were also reported by Preziuso and Russo (2004) and Bures and Barton (2012). But these results have not been associated with findings reported by Funghi et al. (1994), who found that L value decreased as slaughter age increased. The color of meat from meat quality traits has an important role for a consumer's purchase decisions and is affected by a number of pre-slaughter and post-slaughter factors. Meat lightness is often inversely correlated to heme iron content, which increases as slaughter age increases (Bures and Barton, 2012; Mancini and Hunt, 2005; Chambaz et al., 2003). Results from this study were in accordance with findings of Bures and Barton (2012), who stated that lightness was effected by slaughtering age of animals, and the color of meat became darker as slaughter age increased.

**Figure 1:**
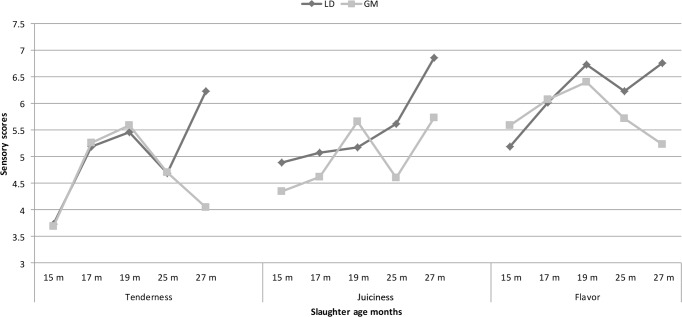
Changes in tenderness, juiciness and flavor scores of LD and GM muscles depending on the different slaughter age.

The chemical composition of the meat is presented in Table 2. Determining chemical composition of meat is necessary for assessing nutritive value of meat. The effects of slaughter age and muscle type on the proportions of dry matter, fat, ash and protein were not significant. It is known that intramuscular fat may positively influence some sensory characteristics such as tenderness, juiciness, flavor, etc. (Bures and Barton, 2012). Although the chemical composition parameters were not affected by slaughter age, muscle type and slaughter age × muscle type interaction, dry matter, fat and ash contents increased as slaughter age increased. Similar results were also reported by Van Koevering et al. (1995), Dubeski et al. (1997), and Bures and Barton (2012).

The sensory characteristics of beef are presented in Table 2. Meat tenderness is one of the most important textural traits from the meat quality characteristics and has the greatest effect on consumer satisfaction of meat. The effect of slaughter age on tenderness, juiciness, acceptability, number of chews and WBS values was determined as significant (p<0.05; p<0.01). Tenderness, juiciness, flavor intensity and acceptability increased up to 19 months of age, and then increase in slaughter age resulted in lower tenderness, flavor intensity and acceptability scores. This result is an indicator related to a negative influence of increased age on beef meat texture. The amount of intramuscular fat increase, depending on the increase of slaughter age of the animal, is likely to contribute to improvement of the sensory panel ratings (Maltin et al., 1998). Possible explanations for the reduced meat texture obtained from the muscles of older animals are the smaller extent of post-mortem proteolysis, the bigger size of muscle fibers (Crouse et al., 1991; Tornberg et al., 1994) and a lower solubility of collagen. Tenderness in meat is also associated with the amount of collagen in muscles. This result may be attributed to the high relationship among the intramuscular fat, tenderness and flavor density. Results from the present study were similar with findings reported by some researchers (Cross et al., 1984; Renand et al., 2001; Bures and Barton, 2012; Dominguez et al., 2015). Contrary to findings from the present study, some authors reported that slaughter age did not have any significant effect on sensory panel ratings (Riley et al., 1986; Sami et al., 2004; Mojto et al., 2009). As shown in Table 2, the highest scores for sensory evaluation were obtained from 19-month-old animals.

Differences between LD and GM muscles in terms of sensory characteristics except for flavor and juiciness were not significant. Juiciness and flavor scores determined for LD muscle were greater (p<0.01; p<0.05) than those of GM muscle. The muscle type was a significant variation source for sensory traits and WBS force values.

The slaughter age × muscle type interaction had a significant effect on sensory characteristics except for acceptability and number of chews before swallowing (NCBS). Figure 1 shows the changes in the sensory panel scores at different slaughter ages. Tenderness and flavor panel scores of LD and GM muscles increased from 15 to 19 months of age and decreased from 19 to 25 months of age. Thereafter during the late slaughter period LD tenderness and flavor scores increased; however sensory scores of GM decreased at the same ages. The juiciness values of LD muscle gradually increased up to 25 months of age and thereafter rapidly increased (Fig. 1). Results for tenderness, flavor and juiciness were similar to findings reported by Nishimura et al. (1999).

**Figure 2:**
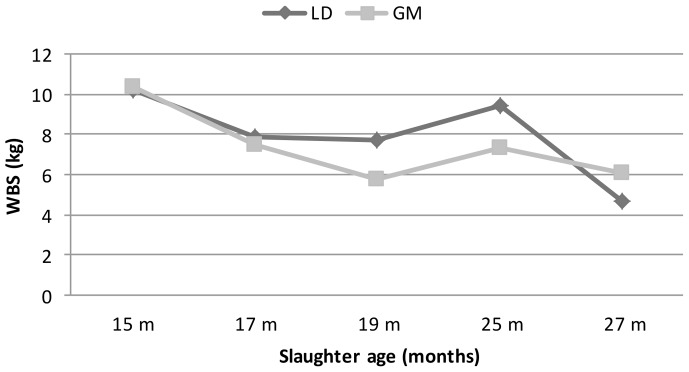
Changes in Warner–Bratzler shear (WBS) force values of LD and GM muscles depending on different slaughter ages.

The highest WBS values were obtained at 15 months of age, and these WBS values decreased as the slaughter age of animals increased. This situation is expressed by the fact that the meat is harder due to insufficient muscle fattening by young animals, and then the muscles become more tender with the increase of intramuscular fat with the advancing ages (Marti et al., 2013; Renand et al., 2001; Dominguez et al., 2015).

**Table 3:** Effect of slaughter age and muscle type on texture of meat (mean ± SEM).

	Slaughter age (A)						Muscle (M)			Significance		
	15 months	17 months	19 months	25 months	27 months		GM	LD		A	M	A×M
TPA												
Hardness (N)	12.26±1.57	9.10±1.48	11.68±1.42	10.55±1.48	11.79±1.91		12.60±1.0	9.56±1.00		ns	${}^{*}$	ns
Cohesiveness	0.62±0.01	0.66±0.01	0.64±0.01	0.66±0.01	0.65±0.01		0.66±0.01	0.64±0.01		ns	ns	ns
Springiness (mm)	0.49±0.03	0.43±0.03	0.52±0.03	0.47±0.03	0.50±0.4		0.50±0.02	0.47±0.02		ns	ns	ns
Gumminess (N)	7.56±0.93	5.98±0.88	7.38±0.84	6.86±0.88	7.62±1.13		8.18±0.59	5.98±0.59		ns	${}^{**}$	ns
Chewiness (N)	3.94±0.59	2.79±0.56	4.02±0.53	3.32±0.56	4.01±0.72		4.29±0.38	2.95±0.38		ns	${}^{**}$	ns
Resilience (N mm)	0.29±0.01	0.31±0.01	0.31±0.01	0.32±0.01	0.32±0.01		0.32±0.01	0.30±0.01		ns	${}^{*}$	ns

ns: non-significant (p>0.05); ${}^{**}$ p<0.01; ${}^{*}$ p>0.05; SEM: standard error of means.

It has been observed that the WBS values of LD and GM muscles at different slaughter ages are similar to the sensory scores (Fig. 2). The WBS values of LD and GM muscles decreased up to 19 months of age and then increased gradually until 25 months of age. However, the lowest shear values in LD were obtained in 27 months of age in parallel with sensory scores. These results were similar to the findings of Nishimura et al. (1999). Also, there was a demonstrable relationship between the WBS and sensory panel scores ($r=-0.65^{**}$ between WBS and tenderness; $r=-0.57^{**}$ between WBS and juiciness; $r=-0.53^{**}$ between WBS and flavor; $r=-0.61^{**}$ between WBS and acceptability; $r=0.76^{**}$ between WBS and NCBS).

Cooking yield is an indicator of meat quality: the higher the cooking yield, the better the tenderness, juiciness and overall acceptability (Amha, 2006). The slaughter age and muscle type were a significant source of variation (Table 2). The lowest cooking yield was obtained at 15 and 17 months of age, while the highest cooking yield was obtained in 27-month-old animals. The cooking yield values of muscles increased in parallel as slaughter age of animals increased. Similarly, some researchers (Preziuso and Russo, 2004; Franco and Lorenzo, 2014; Dominguez et al., 2015) reported that LD muscle had higher cooking yield value than that of the GM muscle.

Differences with regard to the textural parameters among the age groups were not noticeable (Table 3). Texture analysis results showed that muscle type such as LD and GM had a significant effect on hardness, gumminess, chewiness and resilience. The textural parameters were not affected by slaughter age × muscle type interaction. Results obtained from the present study were similar to findings reported by Lorenzo et al. (2014) and Dominguez et al. (2015). In contrast to other researchers, Polidori et al. (2015) indicated that older animals had higher texture values than those of younger animals.

## 5 Conclusions

The present study confirmed that meat quality characteristics depend on animal age slaughtered at different weights and muscle types. The effects of slaughter age on some meat quality parameters such as pH, meat color, sensory characteristics and WBS were found to be significant. EAR bulls slaughtered at 19 months of age produced good results with regard to most of the meat quality characteristics. So, delaying the slaughter age of animals from 19 months of age to 25–27 months of age may lead to loss of meat quality and therefore economic losses.

## Data Availability

The data sets are available upon request from the corresponding author.
